# Integrating machining learning and multimodal neuroimaging to detect schizophrenia at the level of the individual

**DOI:** 10.1002/hbm.24863

**Published:** 2019-11-18

**Authors:** Du Lei, Walter H. L. Pinaya, Jonathan Young, Therese van Amelsvoort, Machteld Marcelis, Gary Donohoe, David O. Mothersill, Aiden Corvin, Sandra Vieira, Xiaoqi Huang, Su Lui, Cristina Scarpazza, Celso Arango, Ed Bullmore, Qiyong Gong, Philip McGuire, Andrea Mechelli

**Affiliations:** ^1^ Huaxi MR Research Center (HMRRC), Department of Radiology West China Hospital of Sichuan University Chengdu China; ^2^ Department of Psychosis Studies Institute of Psychiatry, Psychology & Neuroscience, King's College London, De Crespigny Park London UK; ^3^ Department of Neuroimaging Institute of Psychiatry, Psychology, and Neuroscience, King's College London London UK; ^4^ Department of Psychiatry and Neuropsychology School of Mental Health and Neuroscience, Maastricht University Medical Center Maastricht The Netherlands; ^5^ Mental Health Care Institute Eindhoven (GGzE) Eindhoven The Netherlands; ^6^ School of Psychology & Center for neuroimaging and Cognitive Genomics, NUI Galway University Galway Ireland; ^7^ Department of Psychiatry School of Medicine, Trinity College Dublin Dublin Ireland; ^8^ Department of General Psychology University of Padua Padua Italy; ^9^ Child and Adolescent Department of Psychiatry Hospital General Universitario Gregorio Marañon, School of Medicine, Universidad Complutense Madrid, IiSGM, CIBERSAM Madrid Spain; ^10^ Brain Mapping Unit, Department of Psychiatry University of Cambridge Cambridge UK; ^11^ Psychoradiology Research Unit of Chinese Academy of Medical Sciences West China Hospital of Sichuan University Chengdu Sichuan China; ^12^ Department of Radiology Shengjing Hospital of China Medical University Shenyang Liaoning China

**Keywords:** functional connectivity, graph theoretical analysis, machine learning, neuroimaging, schizophrenia

## Abstract

Schizophrenia is a severe psychiatric disorder associated with both structural and functional brain abnormalities. In the past few years, there has been growing interest in the application of machine learning techniques to neuroimaging data for the diagnostic and prognostic assessment of this disorder. However, the vast majority of studies published so far have used either structural or functional neuroimaging data, without accounting for the multimodal nature of the disorder. Structural MRI and resting‐state functional MRI data were acquired from a total of 295 patients with schizophrenia and 452 healthy controls at five research centers. We extracted features from the data including gray matter volume, white matter volume, amplitude of low‐frequency fluctuation, regional homogeneity and two connectome‐wide based metrics: structural covariance matrices and functional connectivity matrices. A support vector machine classifier was trained on each dataset separately to distinguish the subjects at individual level using each of the single feature as well as their combination, and 10‐fold cross‐validation was used to assess the performance of the model. Functional data allow higher accuracy of classification than structural data (mean 82.75% vs. 75.84%). Within each modality, the combination of images and matrices improves performance, resulting in mean accuracies of 81.63% for structural data and 87.59% for functional data. The use of all combined structural and functional measures allows the highest accuracy of classification (90.83%). We conclude that combining multimodal measures within a single model is a promising direction for developing biologically informed diagnostic tools in schizophrenia.

## 1 INTRODUCTION

Schizophrenia is a severe psychiatric disorder, characterized by delusions, hallucinations and disorganized thinking (Hu et al., 2016), which affects about 1% of the world's population (Dhindsa & Goldstein, 2016; Lieberman, Scott Stroup, & Perkins, 2006; Nowak, Sabariego, Switaj, & Anczewska, 2016; Rajji, Miranda, & Mulsant, 2014). Its etiology and neuropathology are not well understood, even though neuroimaging studies have revealed distributed structural and functional brain abnormalities (Karlsgodt, Sun, & Cannon, 2010; Oh et al., 2015; Oh et al., 2017). Schizophrenia is usually diagnosed by a clinical examination carried out by psychiatrists. However, accurate diagnosis can take up to 2 years due to its heterogeneous and fluctuating presentation. Given the importance of providing the right treatment to patients in the early stages of the illness, there is an urgent clinical need for an objective diagnostic test that could be used to detect the illness and reduce the risk of misdiagnosis without the need for a long follow‐up.

Within the field of biological psychiatry, there is growing interest in the application of machine learning (ML) techniques to neuroimaging data for the diagnosis of psychiatric illness (Arbabshirani, Castro, & Calhoun, 2014; Kim, Calhoun, Shim, & Lee, 2016; Orru, Pettersson‐Yeo, Marquand, Sartori, & Mechelli, 2012), and the prediction of disease transition in individuals at clinical high risk (Chung et al., 2018; Koutsouleris et al., 2009; Pettersson‐Yeo et al., 2013). Over the past decade, psychiatric disorders such as schizophrenia have been the focus of much research on automatic diagnosis by the integration of ML and neuroimaging (Squarcina et al., 2017; Valli et al., 2016; Zarogianni, Moorhead, & Lawrie, 2013). The vast majority of the existing studies have applied ML techniques to a single neuroimaging modality including structural magnetic resonance imaging (sMRI) (Borgwardt et al., 2013; Koutsouleris et al., 2015; Schnack et al., 2014), resting‐state functional magnetic resonance imaging (fMRI) (Chyzhyk, Grana, Ongur, & Shinn, 2015; S. Wang et al., 2016) or task‐related fMRI (Bendfeldt et al., 2015; Costafreda et al., 2011). Taken collectively, the accuracies reported in these studies tend to be in the 60–80% range. While a small number of studies have reported higher accuracies using sMRI (96%; Pardo et al., 2006) and resting‐state fMRI (100%; Fekete et al., 2013), these tended to include a small number of subjects (e.g., 18 subjects in total in Pardo et al., 2006 and 28 subjects in total in Fekete et al., 2013) and therefore the reliability of the findings is unclear. Considering that patients with schizophrenia show both structural and functional abnormalities (Fitzsimmons, Kubicki, & Shenton, 2013; Karlsgodt et al., 2010), accuracy might be improved by combining different neuroimaging modalities using a multimodal ML framework. So far, a total of four studies have attempted to do this with the aim of detecting schizophrenia at the level of the individual patient (Du et al., 2012; Ota et al., 2013; Qureshi, Oh, Cho, Jo, & Lee, 2017; Sui et al., 2013). However, the results of these multimodal studies have been inconsistent with accuracies ranging 93–98% (Du et al., 2012), 72–88% (Ota et al., 2013), 99.29% (Qureshi et al., 2017), and 79% (Sui et al., 2013) possibly because of the use of small samples (range: 25–72) and different recruitment criteria, possible because of the use of small samples (range: 25–72) and different recruitment criteria. Therefore, it is unclear to what extent multimodal integration can boost the accuracy of classification of schizophrenia.

In this study, we aimed to classify patients with schizophrenia and healthy controls by combining structural (sMRI) and resting‐state functional (rs‐fMRI) data. For sMRI, gray matter and white matter volume were extracted and used as input for classification, whereas for rs‐fMRI we used two most widely used resting‐state metrics, amplitude of low‐frequency fluctuation (ALFF) and regional homogeneity (ReHo). ALFF captures fluctuations in spontaneous, low‐frequency oscillations; in contract ReHo reflects the temporal homogeneity of regional BOLD signals regardless of their intensities. Consequently, these two measures can be used to extract different types of information from the BOLD signal during the resting state. In addition, in light of current neurobiological models of schizophrenia as a dysconnectivity syndrome (Lynall et al., 2010; van den Heuvel, Mandl, Stam, Kahn, & Hulshoff Pol, 2010; Yu et al., 2011) and recent advances in the application of graph‐based theoretical approaches to the human brain (E. Bullmore & Sporns, 2009; E. T. Bullmore & Bassett, 2011), we extracted connectome‐wide based metrics from the sMRI (H. Wang, Jin, Zhang, & Wang, 2016) and rs‐fMRI data (J. Wang, Zuo, & He, 2010) and used these as additional inputs for classification.

In order to assess the reliability of the findings, we used five independent datasets resulting in a total sample of 295 patients with schizophrenia and 452 healthy controls. Each dataset comprised high‐resolution T1‐weighted images and rs‐fMRI, allowing us to examine classification accuracy for each modality as well as their integration. Because schizophrenia is considered to involve structural as well as functional brain abnormalities (Karlsgodt et al., 2010; Oh et al., 2015; Oh et al., 2017), we hypothesized that (a) both structural and functional data would allow single‐subject classification with statistically significant accuracy. In addition, given the existing evidence for both regional and network‐level alterations in schizophrenia (Y. Liu et al., 2008; Micheloyannis et al., 2006; Shen, Wang, Liu, & Hu, 2010), we hypothesized that, within each modality, (b) the combination of voxel‐wise images and connectome‐wide based matrices would be superior to the use of either voxel‐wise images or connectome‐wide based matrices by themselves. Finally, based on (a) and (b), we hypothesized that combining all structural and functional measures within a multimodal, multimeasure model would lead to the highest accuracy of classification.

## 2 MATERIALS AND METHODS

### 2.1 Participants

We used five datasets, each including patients with a diagnosis of schizophrenia and healthy controls. The combination of the five datasets yielded a total sample size of 747, including 295 patients with schizophrenia and 452 healthy controls. The demographic and clinical characteristics of the two groups for each dataset are presented in Table 1.

**Table 1:** Demographic and clinical characteristics of participants^a^

	Dataset 1		Dataset 2		Dataset 3		Dataset 4		Dataset 5	
	SCZ	CON	SCZ	CON	SCZ	CON	SCZ	CON	SCZ	CON
Sample size	68	72	56	132	49	63	32	83	90	102
Disease stage	EST^b^	–	EST	–	EST	–	EST	–	FE	–
Age (years)	38.10 ± 14.13	35.87 ± 11.74	36.16 ± 8.52	30.99 ± 8.62	29.02 ± 6.39	29.60 ± 10.59	40.94 ± 10.90	28.09 ± 8.98	24.31 ± 7.77	30.56 ± 15.21
Gender (M/F)	55/13	51/21	42/14	69/63	38/11	25/38	24/8	38/45	33/57	49/53
Handedness (R/L/B)	56/10/2	69/1/2	NA	NA	40/7/2	54/7/2	32/0/0	83/0/0	90/0/0	102/0/0
Education (years)	NA	NA	NA	NA	16.61 ± 1.99	17.38 ± 2.00	14.72 ± 4.41	17.73 ± 3.28	12.13 ± 3.21	12.27 ± 3.18
Medication (An/Dn)	68/0	NA	45/4^c^	NA	48/1	NA	23/5^d^	NA	0/90	NA
PANSS total	58.78 ± 14.35^e^	NA	NA	NA	44.16 ± 12.40	NA	NA	NA	96.46 ± 17.02^f^	NA
PANSS positive	14.36 ± 4.78^e^	NA	NA	NA	10.10 ± 4.43	NA	NA	NA	26.17 ± 5.40^f^	NA
PANSS negative	15.00 ± 5.36^e^	NA	NA	NA	10.81 ± 5.29	NA	NA	NA	17.90 ± 7.18^f^	NA
PANSS general	29.42 ± 8.55^e^	NA	NA	NA	23.24 ± 5.49	NA	NA	NA	48.05 ± 9.09^f^	NA
SAPS	NA	NA	23.16 ± 17.00^g^	NA	NA	NA	7.58 ± 12.28^h^	NA	NA	NA
SANS	NA	NA	28.30 ± 16.14^g^	NA	NA	NA	13.33 ± 17.85^h^	NA	NA	NA

Abbreviations: SCZ, schizophrenia; CON, control; EST, established; FE, first episode; PANSS, Positive and Negative Syndrome Scale; SAPS, Scale for the Assessment of Positive Symptoms; SANS, Scale for the Assessment of Negative Symptoms; M, male; F, female; R, right; L, left; B, ambidextrous; An, antipsychotic medication, Dn, Drug‐naïve, NA, not available.

a Data are presented as mean ± *SD*.

b Patients were diagnosed with established schizophrenia if duration of illness was more than 24 months.

c Data available for 49 of 56 patients.

d Data available for 28 of 32 patients.

e Data available for 50 of 68 patients.

f Data available for 88 of 90 patients.

g Data available for 50 of 56 patients.

h Data available for 24 of 32 patients.

Dataset 1 was obtained from the Neuroimaging Informatics Tools and Resources Clearinghouse (NITRC) website and was provided by the Centers of Biomedical Research Excellence (COBRE; http://fcon_1000.projects.nitrc.org/indi/retro/cobre.html). In this dataset, a diagnosis of schizophrenia was made using the Structured Clinical Interview for DSM Disorders (SCID; Diagnostic and Statistical Manual of Mental Disorders, DSM‐IV) (M. B First et al., 2012). Exclusion criteria included confirmed or suspected pregnancy, any history of neurological disorders and a history of intellectual disability. Written informed consent was obtained from participants according to institutional guidelines at the University of New Mexico.

Dataset 2, acquired as part of the UCLA Consortium for Neuropsychiatric Phenomics LA5c Study, was obtained from the OpenfMRI database (accession number: ds000030). All patients underwent a semistructured assessment with the Structured Clinical Interview for the Diagnostic and Statistical Manual of Mental Disorders, Fourth Edition (DSM‐IV) (Michael B First, Frances, & Pincus, 2004). Exclusion criteria included left‐handedness, pregnancy, history of head injury with loss of consciousness or cognitive sequelae, or other contraindications to scanning (Poldrack et al., 2016). After receiving a verbal explanation of the study, participants gave written informed consent following procedures approved by the Institutional Review Boards at UCLA and the Los Angeles County Department of Mental Health.

Dataset 3 was acquired at Maastricht University, The Netherlands. Patients were recruited through clinicians working in selected representative geographic areas in the Netherlands and Belgium. Diagnosis of schizophrenia was based on DSM‐IV criteria (American Psychiatric Association, 2000), assessed with the Comprehensive Assessment of Symptoms and History (CASH) interview (Andreasen, Flaum, & Arndt, 1992). Exclusion criteria included confirmed or suspected pregnancy, any history of neurological disorders, a history of intellectual disability and/or a history of substance abuse/dependence within the last 12 months. The ethics committee of Maastricht University approved the study, and all the participants gave written informed consent in accordance with the committee's guidelines and the Declaration of Helsinki.

Dataset 4 was acquired in Dublin and scanned at the Trinity College Institute of Neuroscience as part of a Science Foundation Ireland‐funded neuroimaging genetics study (“A structural and functional MRI investigation of genetics, cognition and emotion in schizophrenia”). Patients with confirmed DSM‐IV diagnosis of schizophrenia were recruited through local clinical services. Exclusion criteria included confirmed or suspected pregnancy, any history of neurological disorders or intellectual disability and substance misuse in the preceding 3 months. Participants provided written, informed consent in accordance with local ethics committee guidelines.

Dataset 5 was acquired at the West China Hospital of Sichuan University, Chengdu, China. An initial diagnosis of schizophrenia and duration of illness were determined by consensus between two experienced psychiatrists, using the Structured Clinical Interview for DSM‐IV (SCID)‐Patient Version (American Psychiatric Association, 2000). In addition, diagnosis of schizophrenia was confirmed for all the patients at 1‐year follow‐up. Exclusion criteria were the existence of a neurological disorder or other psychiatric disorders, alcohol or drug abuse (DSM‐IV), pregnancy, and any chronic physical illness such as a brain tumor, hepatitis, or epilepsy, as assessed by clinical evaluations and medical records. The study was approved by the ethics committee of West China Hospital, and written informed consent was obtained from all participants.

### 2.2 MRI data acquisition

At each site, the high‐resolution three‐dimensional T1‐weighted images and rs‐fMRI images were acquired. Dataset 1 was acquired using a 3T Siemens scanner. The sequence parameters were as follows: repetition time/echo time (TR/TE) = 2530/1.64 ms, flip angle (FA) = 7°, 256 axial slices with slice, thickness = 1 mm, field of view (FOV) = 25.6 × 25.6 cm^2^ and data matrix = 256 × 256, voxel size = 1 × 1× 1 mm^3^. Dataset 2 was acquired using a 3 T Siemens scanner. The sequence parameters were as follows: repetition time/echo time/inversion time (TR/TE/TI) = 2530/3.31/1100 ms, flip angle (FA) = 7°, 256 axial slices with slice, thickness = 1 mm, field of view (FOV) = 25.6 × 25.6 cm^2^ and data matrix = 256 × 256, voxel size = 1 × 1× 1 mm^3^. Dataset 3 was acquired using a 3 T Siemens Magnetom Allegra head scanner. The sequence parameters were as follows: repetition time/echo time/inversion time (TR/TE/TI) = 2250/2.6/900 ms, flip angle (FA) = 8°, 192 axial slices with slice, thickness = 1 mm, field of view (FOV) = 25.6 × 25.6 cm^2^ and data matrix = 256 × 256, voxel size = 1 × 1× 1 mm^3^. Dataset 4 was acquired using a 3T Philips Intera Achieva scanner. The sequence parameters were as follows: repetition time/echo time (TR/TE) = 8.4/3.8 ms, flip angle (FA) = 8°, 180 axial slices with slice, thickness = 0.9 mm, field of view (FOV) = 23 × 23 cm^2^ and data matrix = 256 × 256, voxel size = 0.9 × 0.9 × 0.9 mm^3^. Dataset 5 was acquired using a 3T GE scanner (EXCITE; General Electric, Milwaukee, Wisconsin). The sequence parameters were as follows: repetition time/echo time (TR/TE) = 8.5/3.4 ms, flip angle (FA) = 12°, 156 axial slices with slice, thickness = 1 mm, field of view (FOV) = 24 × 24 cm^2^ and data matrix = 256 × 256. The final matrix of T1‐weighted images was automatically interpolated in plane to 512 × 512, which yields an in‐plane resolution of 0.47 × 0.47 mm^2^.

At each site, the same scanner used to collect the high‐resolution three‐dimensional T1‐weighted images was also employed to acquire the rs‐fMRI images. Dataset 1 was acquired by repetition time/echo time (TR/TE) = 2000/30 ms; flip angle = 90°; 33 axial slices per volume; voxel size = 3.75 × 3.75 × 4.55 mm^3^; number of volumes = 150. Dataset 2 was acquired by repetition time/echo time (TR/TE) = 2000/30 ms; flip angle = 90°; 34 axial slices per volume; voxel size = 3 × 3 × 4 mm^3^; number of volumes = 152. Dataset 3 was acquired by repetition time/echo time (TR/TE) = 1500/30 ms; flip angle = 90°; 27 axial slices per volume; voxel size = 3.5 × 3.5 × 5.2 mm^3^; number of volumes = 200. Dataset 4 was acquired by repetition time/echo time (TR/TE) = 2000/30 ms; flip angle = 90°; 35 axial slices per volume; voxel size = 3.5 × 3.5 × 3.5 mm^3^; number of volumes = 180. Dataset 5 was acquired by repetition time/echo time (TR/TE) = 2000/30 ms; flip angle = 90°; 30 axial slices per volume; voxel size = 3.75 × 3.5 × 5 mm^3^; number of volumes = 200.

### 2.3 MRI data analysis

**Figure 1:**
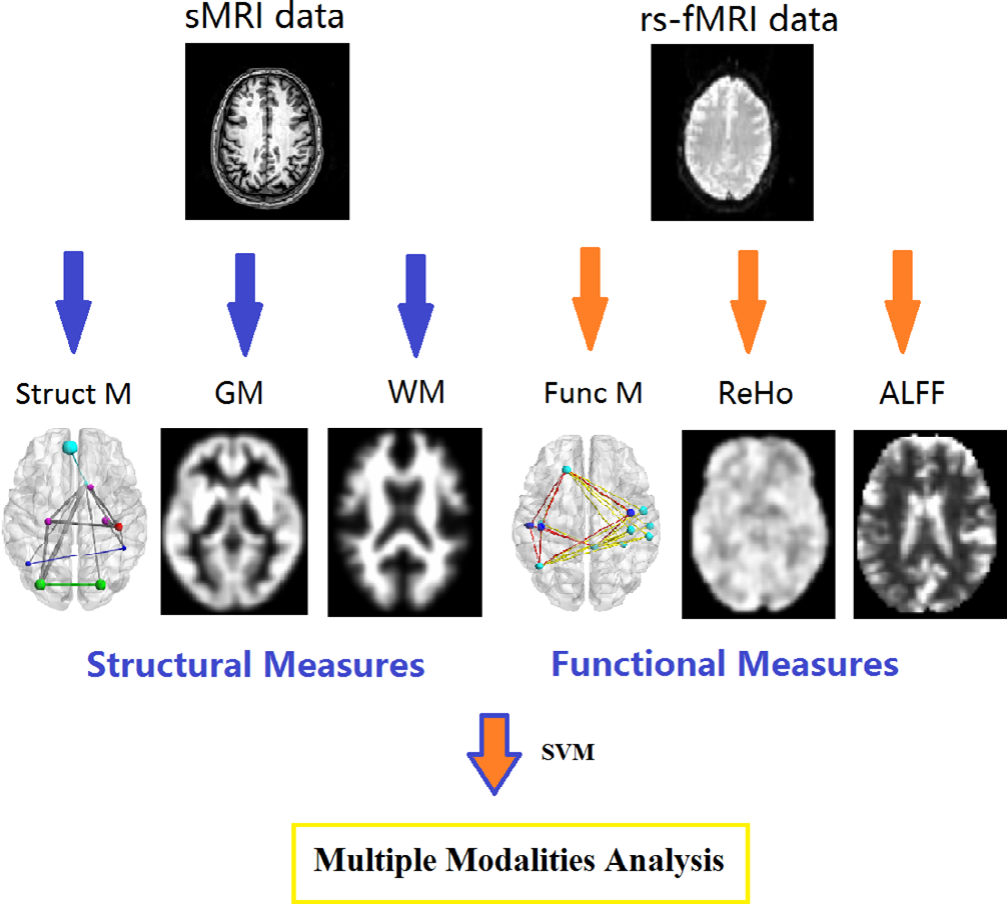
Overview of the employed classification approach. Overview of the classification approach employed to estimate the diagnostic value of fMRI and rs‐fMRI data. ALFF, amplitude of low‐frequency fluctuation; Func M, functional connectivity matrix; GM, gray matter; ReHo, regional homogeneity; rs‐fMRI, resting‐state functional MRI; sMRI, structural MRI; Struct M, structural covariance matrix; WM, white matter

Six individual measures were analyzed using support vector machine, including structural covariance matrix, gray matter and white matter volume, which are extracted from sMRI data, and functional connectivity matrix, ALFF and ReHo, which were extracted from rs‐fMRI data (Figure 1).

#### 2.3.1 Extraction of voxel‐wise measures from structural data

Structural images were processed using Statistical Parametric Mapping software (SPM8; http://www.fil.ion.ucl.ac.uk/spm). In brief, individual structural images were first segmented into gray matter (GM) and white matter (WM) using the unified segmentation model (Ashburner & Friston, 2005). The resulting GM and WM maps were then normalized to the Montreal Neurological Institute (MNI) space using a high‐dimensional “DARTEL” approach and subjected to nonlinear modulation to compensate for spatial normalization effects. Finally, the GM and WM data were resampled to 1.5 mm^3^ voxels and spatially smoothed (Gaussian kernel with a full width at half maximum of 6 mm). The preprocessed GM and WM volume images would then be used as features for the ML analyses.

#### 2.3.2 Extraction of covariance matrix from structural data

Following the preprocessing of the structural data, we constructed large‐scale morphological brain networks for each participant based on their GM volume images. First, to define the network nodes, we parcellated the brain into different regions of interests (ROIs) in terms of automated anatomical labeling (AAL) 90 atlas. Next, to estimate internodal network edges, we utilized a Kullback–Leibler divergence‐based (Kullback & Leibler, 1951) similarity measure to quantify morphological connectivity between two regions (Kong et al., 2014), which generated a 90 × 90 morphological brain networks matrix for each individual (H. Wang, Jin, et al., 2016).

#### 2.3.3 Functional data preprocessing

Image preprocessing was performed using SPM8 and DPARSF software (http://restfmri.net/forum/dparsf_v2_2) (Chao‐Gan & Yu‐Feng, 2010). The first 10 time points were discarded to minimize the impact of the instability in the initial MRI signal. The remaining images were corrected for intravolume acquisition time delay. To minimize the potential impact of head motion artifacts—a recognized challenge in rs‐fMRI analyses (Power, Barnes, Snyder, Schlaggar, & Petersen, 2012; Satterthwaite et al., 2013), we applied Friston 24‐parameter correction (Yan, Craddock, He, & Milham, 2013) and the “head motion scrubbing” method proposed by Power and colleagues (Power et al., 2014) to ensure that motion artifacts were not contributing to the group differences we observed. For each participant, volumes with framewise displacement (FD) greater than 0.5 mm were identified and excluded. After these corrections, the images were spatially normalized to a 3 × 3 × 3 mm^3^ MNI 152 template and then linearly detrended and temporally bandpass filtered (0.01–0.08 Hz) to remove low‐frequency drift and high‐frequency physiological noise. Finally, the global signal, the white matter signal, the cerebrospinal fluid (CSF) signal and the motion parameters (1.5 translational and 1.5 rotational parameters) were regressed out (Fox, Zhang, Snyder, & Raichle, 2009). None of the subjects included in the present investigation showed excessive head motion during scanning (defined as translational movement >1.5 mm and/or rotation >1.5°).

#### 2.3.4 Extraction of voxel‐wise measures from functional data

ReHo maps were extracted from the preprocessed images using DPARSF software. After removing linear trends in the unsmoothed images and applying a bandpass filter (0.01 < *f* < 0.08 Hz) to the data to reduce low‐frequency drift and high‐frequency respiratory and cardiac noise, ReHo maps were generated by calculating the concordance of Kendall's coefficient (with values ranging from 0 to 1) of the time series of a given voxel with those of its 26 nearest neighbors. The ReHo value of each voxel was then standardized by dividing this value by the global (within the brain) mean ReHo value.

The ALFF was also calculated using DPARSF software. After application of a band‐pass filter (0.01–0.08 Hz) and removal of linear trends, the time series were transformed to the frequency domain using fast Fourier transforms (FFTs). The square root of the power spectrum was calculated and was then averaged across 0.01–0.08 Hz for each voxel. This averaged square root was referred to as ALFF. Finally, the ALFF of each voxel was standardized by dividing it by the global (within the brain) mean ALFF value for further statistical analysis.

#### 2.3.5 Extraction of functional connectivity matrices from functional data

The graph theoretical network analysis was performed using GRETNA software (http://www.nitrc.org/projects/gretna/) (J. Wang et al., 2015). First, the whole brain was divided into 90 cortical and subcortical ROI—each representing a network node—using the AAL atlas. Next, to define the edges of the network, we extracted the mean time series of each region and calculated Pearson's correlations of the mean time series between all pairs of nodes. This process resulted in a 90 × 90 weighted correlation matrix for each subject.

### 2.4 Support vector machine

We performed all machining learning analyses using Python programming language, and made the scripts publicly available on https://github.com/Warvito/integrating‐multi‐modal‐neuroimaging. For each dataset, we used support vector machine (SVM) (Cortes & Vapnik, 1995) to perform single‐subject classification. SVM maps the input vectors to a feature space using a set of mathematical functions known as kernels. In this feature space, the model finds the optimum separation surface that can maximize the margin between different classes within a training dataset. Once the separation surface is determined, it can be used to predict the class of new observations using an independent testing dataset. Here a linear kernel was preferred to a nonlinear one to minimize the risk of overfitting. The model was based on LIBSVM (Chang & Lin, 2011) and implemented by the Scikit‐Learn library (Pedregosa et al., 2012).

During the multiple measure analysis, we combined the SVM predictions of single measures using a weighted averaging method (i.e., soft voting), as a previous study had reported that this method appeared to be slightly more effective than either sum of kernels or multikernel learning (MKL) (Pettersson‐Yeo et al., 2014). In this approach, we first trained each SVM using a single measure; this allowed us to estimate the likelihood of an individual belonging to the patient or control group (the likelihood was calculated using Scikit‐Learn library default method). Then, we calculated the weighted probabilities of each specific measure by multiplying its predicted probabilities by a coefficient (see Section 2.4.1 for how this was optimized). Finally, we calculated the average of the predicted weighted probabilities, and the group with the highest score was defined as the predicted class for a given subject.

#### 2.4.1 Evaluation of the support vector machines

For each SVM model, we used an independent set of individuals to perform a nonbiased assessment the performance. Specifically, a 10‐fold stratified cross‐validation scheme was used to separate the original samples (of each dataset) in 10 nonoverlapping partitions. In each iteration of the scheme, one partition was considered as the independent test set (where the performance metric is calculated), and the remaining subjects were defined as the training sample.

Within each training set, we performed an internal cross‐validation (i.e., 10‐fold stratified nested cross‐validation) to select the optimal set of hyperparameters of the ML models. The linear SVM has only one hyperparameter (the soft margin parameter *C*) that controls the trade‐off between reducing training errors and having a larger separation margin. This parameter was optimized performing a grid search in the following range of values: *C* = 10^−3^, 10^−2^, 10^−1^, 1, 10^1^, 10^2^, 10^3^, 10^4^. At the end of this internal cross‐validation, we had the optimum *C* value for each input measure.

When we performed a multiple measure analysis, after the grid searches for the *C* parameter, a second nested cross‐validation was performed to optimize the coefficient of each specific measure for the soft voting. Each coefficient was evaluated using a grid search with a coefficient search space assuming an integer value between 1 and 10. This second nested cross‐validation was also performed using a scheme of 10‐fold stratified cross‐validation. In both nested cross‐validation, the highest mean balanced accuracy of the model was used to find the best hyperparameter value.

After these nested cross‐validation steps, an SVM model with the optimal set of values of the hyperparameters was trained using the whole training set. Its performance was assessed on the test set in terms of balanced accuracy, specificity, and sensitivity. The reported balanced accuracy, specificity, and sensitivity are the mean values of the metrics calculated on each partition of the cross‐validation scheme. Statistical significance was estimated using permutation testing (1,000 permutations). The whole training process was performed 1,000 times with the subject labels permuted. The *p*‐values were then obtained by dividing the number of times that the permuted version was better than the original performance by the number of permutations.

#### 2.4.2 Controlling for age and sex effects

For each measure of brain function (i.e., whole brain images, connectome‐wide matrices or graph‐based metrics) in each dataset, we build a regression model that represented how the measure varied with age and sex, and subtracted age‐ and gender‐related variance from the actual measures. This was done using the Gaussian process regression method and kernel function that were used in a previous investigation (Kostro et al., 2014), with the regression model based on control subjects only. The resulting residuals would then be used as features for the ML analyses.

#### 2.4.3 Using different datasets for training and testing

In this study, the five datasets were acquired using different scanners and different scanning parameters. Because site‐related differences are likely to be larger than differences between patients and controls, we did not expect it would be feasible to use subjects from one site as training sample and subjects from another site as testing sample. Nevertheless, we explored the feasibility of this by using different datasets as training and testing data, and 10‐fold cross‐validation was used to assess the performance of the model.

#### 2.4.4 Which brain regions provided the greatest contribution to single‐subject classification?

In order to explore which brain regions contributed to single‐subject classification, we computed the mean absolute value of the weights of the multimodal and multimeasure model, which yielded the highest balanced accuracy across the five datasets. For matrix‐based measures (i.e., structural covariance and functional connectivity), we computed the mean absolute value of the weights for each brain region with the remaining 89 regions across the different folds of the cross‐validation. In contrast, for voxel‐wise measures (GM, WM, ReHo, and ALFF), we computed the mean absolute values of the weights of the model across the different folds of the cross‐validation, and then we used a template mask based on the AAL atlas to extract the value of weight for each brain regions. The 10 brain regions with the highest mean values, computed by averaging the weights across the five datasets, are going to be reported.

## 3 RESULTS

### 3.1 Classification performance

*Individual measures*. Seven hundred and forty‐seven participants (295 patients with schizophrenia; 452 healthy controls) from five different research sites were included in the analysis. The balanced accuracies, sensitivities, specificities, and *p*‐values for the single‐subject classification of patients and healthy controls are reported in Table 2. It can be seen that the balanced accuracy reached statistical significance for each of our six measures of interest, namely GM, WM, structural covariance matrix, ReHo, ALFF, and functional connectivity matrix. This finding was replicated across each of the five independent datasets. When the results for the five datasets were averaged, the mean balanced accuracy was 75.11% for structural covariance matrix, 78.49% for GM, 73.92% for WM, 83.07% for functional connectivity matrix, 82.18% for ReHo, and 83% for ALFF. This pattern of results suggests that, overall, functional data allow higher accuracy of classification than structural data (mean 82.75% vs. 75.84%).

**Table 2:** Single‐subject classification of patients with schizophrenia and healthy controls across different measures

Measures		BAC (%)	SEN^a^ (%)	SPEC^a^ (%)	*P* value^b^
Struct M	Dataset 1	77.26	61.67	92.86	<.001
	Dataset 2	60.88	34.67	87.09	<.001
	Dataset 3	81.50	63.00	100.00	<.001
	Dataset 4	81.25	65.00	97.50	<.001
	Dataset 5	74.65	61.11	88.18	<.001
	Average	75.11	57.09	93.13	
	Pooled	55.94	45.73	66.14	=.02
	Stratified	56.74	44.79	68.70	<.001
GM	Dataset 1	83.45	68.33	98.57	<.001
	Dataset 2	67.26	40.67	93.85	<.001
	Dataset 3	81.17	64.00	98.33	<.001
	Dataset 4	84.17	68.33	100.00	<.001
	Dataset 5	76.38	56.67	96.09	<.001
	Average	78.49	59.60	97.37	
	Pooled	59.11	41.43	76.79	<.001
	Stratified	58.84	43.26	74.42	<.001
WM	Dataset 1	73.36	52.62	94.64	<.001
	Dataset 2	61.59	29.33	93.85	<.001
	Dataset 3	77.04	55.50	98.57	<.001
	Dataset 4	77.71	56.67	98.75	<.001
	Dataset 5	79.92	66.67	93.18	<.001
	Average	73.92	52.16	95.80	
	Pooled	56.31	40.85	71.76	=.003
	Stratified	55.68	40.05	71.31	=.004
Struct M + GM + WM	Dataset 1	87.59	82.14	93.04	<.001
	Dataset 2	64.40	35.67	93.13	<.001
	Dataset 3	86.92	80.50	93.33	<.001
	Dataset 4	86.94	75.00	98.89	<.001
	Dataset 5	82.32	80.00	84.64	<.001
	Average	81.63	70.66	92.61	
	Pooled	58.39	51.67	65.11	<.001
	Stratified	58.17	50.67	65.66	<.001
Func M	Dataset 1	88.21	79.29	97.14	<.001
	Dataset 2	76.35	55.00	97.69	<.001
	Dataset 3	85.25	70.50	100.00	<.001
	Dataset 4	81.87	65.00	98.75	<.001
	Dataset 5	83.65	71.11	96.18	<.001
	Average	83.07	68.18	97.952	
	Pooled	58.58	45.15	72.01	<.001
	Stratified	58.00	45.86	70.15	<.001
ReHo	Dataset 1	84.07	68.14	100.00	<.001
	Dataset 2	79.46	62.00	96.92	<.001
	Dataset 3	83.25	66.50	100.00	<.001
	Dataset 4	82.36	65.83	98.89	<.001
	Dataset 5	81.78	65.56	98.00	<.001
	Average	82.18	65.61	98.76	
	Pooled	54.42	35.50	73.34	=.039
	Stratified	54.25	35.7	72.8	<.001
ALFF	Dataset 1	86.90	73.81	100.00	<.001
	Dataset 2	78.62	63.33	93.90	<.001
	Dataset 3	82.92	67.50	98.33	<.001
	Dataset 4	86.25	72.50	100.00	<.001
	Dataset 5	80.32	65.56	95.09	<.001
	Average	83.00	68.54	97.46	
	Pooled	57.49	39.52	75.47	<.001
	Stratified	56.75	38.98	74.52	<.001
Func M + ReHo + ALFF	Dataset 1	92.14	88.57	95.71	<.001
	Dataset 2	78.93	64.67	93.19	<.001
	Dataset 3	87.64	81.00	94.29	<.001
	Dataset 4	90.28	84.17	96.39	<.001
	Dataset 5	88.97	86.67	91.27	<.001
	Average	87.59	81.02	94.17	
	Pooled	57.73	52.66	62.40	<.001
	Stratified	57.96	52.44	63.49	<.001
Struct M + GM + WM + Func M + ReHo + ALFF	Dataset 1	95.71	94.29	97.14	<.001
	Dataset 2	79.74	63.33	96.15	<.001
	Dataset 3	94.29	90.00	98.57	<.001
	Dataset 4	92.92	85.83	100.00	<.001
	Dataset 5	91.50	90.00	93.00	<.001
	Average	90.83	84.69	96.97	
	Pooled	59.38	52.38	66.39	<.001
	Stratified	58.27	52.33	64.21	<.001

Abbreviations: ALFF, amplitude of low‐frequency fluctuation; BAC, balanced accuracy; Func M, functional connectivity matrix; GM, gray matter; Pooled, pooled the five datasets; ReHo, regional homogeneity; SEN, sensitivity; SPEC, specificity; Stratified, site‐stratified cross‐validation; Struct M, structural covariance matrix; WM, white matter.

a Sensitivity and specificity were computed considering the patient group as the positive class.

b Statistical significance was estimated using the permutation method (1,000 permutations).

#### 3.1.1 Multimeasure integration within a single modality

When combining the three measures extracted from structural images, namely GM, WM, structural covariance matrix, the balanced accuracy reached statistical significance for each of the five datasets (Table 2). Here the mean balanced accuracy of the five datasets was 81.63%. Likewise, when combining the three measures extracted from functional images, namely ReHo, ALFF and functional connectivity matrix, the balanced accuracy reached statistical significance for each of the five datasets. Here the mean balanced accuracy of the five datasets was 87.59%. This pattern of results suggests that, within structural or functional modalities, the combination of connectome‐wide based matrices and voxel‐wise images allows a marginally higher accuracy of classification than the use of either connectome‐wide based matrices or voxel‐wise images alone.

#### 3.1.2 Multimodal and multimeasure integration

When combining all measures across structural and functional modalities, balanced accuracies reached statistical significance for each of the five datasets (Table 2). Here the averaging of the results across the five datasets resulted in the highest accuracy of classification (90.83%). Therefore, multimodal integration resulted in higher accuracy of classification than the use of single modalities, either structural (90.83% vs. 81.63%) or functional (90.83% vs. 87.59%).

#### 3.1.3 Using different datasets for training and testing

The results are reported in Table 3; it can be seen that, as we expected, an algorithm developed using one dataset does not perform well when applied to other datasets. Therefore, in our manuscript, we have opted to analyze and report the five datasets separately; this allowed us to detect reliable effects that were expressed across the different datasets.

**Table 3:** Single‐subject classification of patients with schizophrenia and healthy controls using different training dataset across different measures

Measures	Training dataset	Dataset 1	Dataset 2	Dataset 3	Dataset 4	Dataset 5
Struct M	Dataset 1		57.60	56.8	63.69	55.95
	Dataset 2	56.05		57.71	56.36	54.41
	Dataset 3	60.58	54.90		54.91	52.71
	Dataset 4	57.48	52.71	54.54		55.75
	Dataset 5	51.76	54.38	57.03	64.78	
GM	Dataset 1		62.26	64.74	63.82	56.31
	Dataset 2	52.98		53.06	54.69	50.62
	Dataset 3	61.07	55.49		52.88	52.19
	Dataset 4	53.76	51.79	56.12		52.22
	Dataset 5	51.55	57.66	56.12	57.21	
WM	Dataset 1		52.19	63.27	64.06	53.04
	Dataset 2	55.88		57.14	55.65	55.62
	Dataset 3	53.68	53.19		51.32	50.62
	Dataset 4	58.25	50.51	58.16		52.78
	Dataset 5	50.78	56.01	55.10	50.00	
Struct M + GM + WM	Dataset 1		65.48	67.46	69.22	59.12
	Dataset 2	50.78		52.04	54.69	51.11
	Dataset 3	68.46	56.03		63.57	53.76
	Dataset 4	57.43	52.68	58.16		52.78
	Dataset 5	52.29	58.17	58.16	51.68	
Func M	Dataset 1		61.63	63.27	57.57	50.62
	Dataset 2	54.41		54.08	59.38	52.22
	Dataset 3	54.45	53.22		59.73	56.11
	Dataset 4	59.56	55.25	55.10		52.22
	Dataset 5	56.62	57.55	52.27	52.25	
ReHo	Dataset 1		56.30	62.93	61.30	51.67
	Dataset 2	50.74		51.02	54.69	50.56
	Dataset 3	56.62	56.01		59.38	51.73
	Dataset 4	57.35	58.17	53.06		50.56
	Dataset 5	52.21	54.73	50.23	53.12	
ALFF	Dataset 1		67.48	62.47	65.14	57.25
	Dataset 2	56.62		52.04	58.17	52.29
	Dataset 3	62.50	57.41		72.10	53.79
	Dataset 4	50.00	54.46	50.00		50.56
	Dataset 5	52.21	54.98	50.00	49.76	
Func M + ReHo + ALFF	Dataset 1		69.45	77.44	65.61	56.5
	Dataset 2	58.09		51.02	59.38	51.18
	Dataset 3	66.22	66.37		66.94	53.53
	Dataset 4	55.15	58.55	53.06		52.22
	Dataset 5	57.35	58.58	50.45	50.72	
Struct M + GM + WM + Func M + ReHo + ALFF	Dataset 1		64.45	72.11	65.14	55.26
	Dataset 2	51.47		50.00	53.12	51.11
	Dataset 3	64.01	58.69		62.26	50.00
	Dataset 4	57.35	52.68	53.06		51.67
	Dataset 5	51.47	56.25	54.08	50.96	

Abbreviations: ALFF, amplitude of low‐frequency fluctuation; Func M, functional connectivity matrix; GM, gray matter; ReHo, regional homogeneity; Struct M, structural covariance matrix; WM, white matter.

#### 3.1.4 Brain regions providing the greatest contribution to single‐subject classification

**Figure 2:**
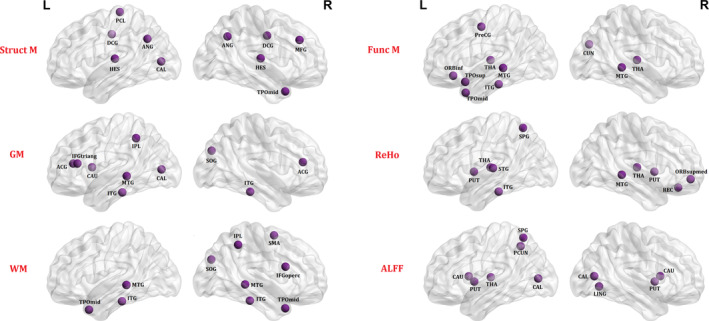
Regions providing the greatest contribution to single‐subject classification. Ten brain regions making the greatest contribution to single‐subject classification for each of our six measures of interest. The nodes were mapped onto the cortical surfaces by using the BrainNet Viewer package (http://www.nitrc.org/projects/bnv). ACG, anterior cingulate and paracingulate gyri; ALFF, amplitude of low‐frequency fluctuation; ANG, angular gyrus; CAL, calcarine; CAU, caudate nucleus; CUN, cuneus; DCG, median cingulate and paracingulate gyri; Func M, functional connectivity matrix; GM, gray matter; HES, Heschl gyrus; IFGoperc, inferior frontal gyrus, opercular part; IFGtriang, inferior frontal gyrus, triangular part; IPL, inferior parietal gyrus; ITG, inferior temporal gyrus; L, left hemisphere; LING, lingual gyrus; MFG, middle frontal gyrus; MTG, middle temporal gyrus; ORBinf, inferior frontal gyrus, orbital part; ORBsupmed, superior frontal gyrus, medial orbital part; PCL, paracentral lobule; PCUN, precuneus; PreCG, Precental gyrus; PUT, putamen; R, right hemisphere; REC, gyrus rectus; ReHo, regional homogeneity; SMA, supplementary motor area; SOG, superior occipital gyrus; SPG, superior parietal gyrus; STG, superior temporal gyrus; Struct M, structural covariance matrix; THA, thalamus; TPOmid, temporal pole: middle temporal gyrus; TPOsup, temporal pole: superior temporal gyrus; WM, white matter

The 10 brain regions with the highest mean values across the five datasets are reported in Table 4 and represented graphically in Figure 2. It can be seen that, within the structural modality, the brain regions contributing to single‐subject classification varied across our three measures of interest. Only the inferior temporal gyrus and middle temporal gyrus were detected consistently across GM and WM measures. In contrast, within the functional modality, the thalamus featured consistently across our three measures of interest. In addition, the inferior temporal gyrus (functional matrices and ReHo), middle temporal gyrus (functional matrices and ALFF) and putamen (ReHo and ALFF) were detected in two of our three measures of interest. Taken collectively, these results suggest that the pattern of regions contributing to single‐subject classification is dependent on the specific structural or functional measure being employed.

**Table 4:** Ten brain regions making the greatest contribution to single‐subject classification across the different measures

Struct M	Dataset 1	Dataset 2	Dataset 3	Dataset 4	Dataset 5	Mean
Median cingulate and paracingulate gyri L	0.0191	0.0383	0.0488	0.0516	0.0184	0.0352
Paracentral lobule L	0.0171	0.0503	0.0453	0.0308	0.0264	0.0340
Heschl gyrus R	0.0197	0.0448	0.0490	0.0323	0.0193	0.0330
Heschl gyrus L	0.0142	0.0496	0.0342	0.0430	0.0239	0.0330
Calcarine L	0.0200	0.0615	0.0348	0.0258	0.0211	0.0326
Median cingulate and paracingulate gyri R	0.0133	0.0507	0.0349	0.0388	0.0240	0.0323
Angular gyrus R	0.0243	0.0470	0.0354	0.0345	0.0196	0.0322
Middle frontal gyrus R	0.0099	0.0722	0.0284	0.0222	0.0264	0.0318
Angular gyrus L	0.0174	0.0596	0.0343	0.0202	0.0208	0.0305
Temporal pole: Middle temporal gyrus R	0.0137	0.0531	0.0348	0.0276	0.0226	0.0304

GM	Dataset 1	Dataset 2	Dataset 3	Dataset 4	Dataset 5	Mean
Superior occipital gyrus R	0.0123	0.0285	0.0279	0.0134	0.0148	0.0194
Calcarine L	0.0069	0.0385	0.0131	0.0129	0.0146	0.0172
Inferior temporal gyrus L	0.0144	0.0157	0.0115	0.0159	0.0131	0.0141
Inferior temporal gyrus R	0.0177	0.0115	0.0129	0.0137	0.0105	0.0132
Middle temporal gyrus L	0.0084	0.0095	0.0127	0.0141	0.0166	0.0123
Inferior parietal L	0.0124	0.0085	0.0090	0.0103	0.0135	0.0108
Anterior cingulate and paracingulate gyri R	0.0226	0.0066	0.0058	0.0078	0.0065	0.0099
Caudate L	0.0011	0.0170	0.0086	0.0121	0.0091	0.0096
Inferior frontal gyrus, triangular part L	0.0212	0.0042	0.0095	0.0101	0.0003	0.0091
Anterior cingulate and paracingulate gyri L	0.0085	0.0043	0.0181	0.0052	0.0033	0.0079

WM	Dataset 1	Dataset 2	Dataset 3	Dataset 4	Dataset 5	Mean
Inferior temporal gyrus R	0.0108	0.0224	0.0250	0.0263	0.0013	0.0171
Temporal pole: Middle temporal gyrus R	0.0023	0.0430	0.0134	0.0059	0.0102	0.0150
Inferior temporal gyrus L	0.0007	0.0154	0.0208	0.0186	0.0135	0.0138
Temporal pole: Middle temporal gyrus L	0.0105	0.0369	0.0118	0.0056	0.0007	0.0131
Superior occipital gyrus R	0.0015	0.0369	0.0147	0.0040	0.0072	0.0129
Middle temporal gyrus L	0.0094	0.0116	0.0143	0.0128	0.0064	0.0109
Inferior frontal gyrus, opercular part R	0.0131	0.0035	0.0188	0.0125	0.0066	0.0109
Middle temporal gyrus R	0.0123	0.0017	0.0086	0.0191	0.0124	0.0108
Inferior parietal R	0.0099	0.0059	0.0121	0.0157	0.0078	0.0102
Supplementary motor area R	0.0228	0.0145	0.0023	0.0054	0.0012	0.0093

Func M	Dataset 1	Dataset 2	Dataset 3	Dataset 4	Dataset 5	Mean
Thalamus L	0.0109	0.0128	0.0115	0.0083	0.0145	0.0116
Thalamus R	0.0114	0.0117	0.0096	0.0086	0.0144	0.0112
Inferior temporal gyrus L	0.0113	0.0101	0.0099	0.0089	0.0151	0.0111
Middle temporal gyrus L	0.0096	0.0130	0.0102	0.0066	0.0151	0.0109
Temporal pole: Middle temporal gyrus	0.0118	0.0115	0.0092	0.0087	0.0131	0.0109
Middle temporal gyrus R	0.0117	0.0122	0.0074	0.0071	0.0151	0.0107
Cuneus R	0.0099	0.0090	0.0115	0.0091	0.0137	0.0106
Temporal pole: Superior temporal gyrus L	0.0094	0.0125	0.0100	0.0071	0.0138	0.0106
Precentral gyrus L	0.0091	0.0107	0.0099	0.0084	0.0131	0.0102
Inferior frontal gyrus, orbital part L	0.0092	0.0116	0.0094	0.0065	0.0145	0.0102

ReHo	Dataset 1	Dataset 2	Dataset 3	Dataset 4	Dataset 5	Mean
Thalamus L	0.1273	0.1553	0.0634	0.1094	0.0871	0.1085
Thalamus R	0.0600	0.1052	0.0581	0.1256	0.0549	0.0808
Gyrus rectus R	0.0545	0.0670	0.0499	0.1184	0.0941	0.0768
Superior temporal gyrus L	0.0693	0.0849	0.0824	0.1046	0.0359	0.0754
Superior parietal gyrus L	0.0470	0.0814	0.0596	0.0776	0.0851	0.0701
Putamen R	0.0122	0.0737	0.0256	0.0439	0.1440	0.0599
Superior frontal gyrus, medial orbital R	0.0542	0.0668	0.0522	0.0400	0.0435	0.0513
Inferior temporal gyrus L	0.0906	0.0371	0.0327	0.0337	0.0456	0.0479
Middle temporal gyrus R	0.0013	0.0321	0.0425	0.0247	0.0560	0.0313
Putamen L	0.0237	0.0148	0.0298	0.0202	0.0428	0.0263

ALFF	Dataset 1	Dataset 2	Dataset 3	Dataset 4	Dataset 5	Mean
Calcarine L	0.0103	0.0270	0.0102	0.0102	0.0225	0.0160
Superior parietal gyrus L	0.0126	0.0150	0.0125	0.0158	0.0159	0.0144
Putamen R	0.0084	0.0124	0.0100	0.0076	0.0292	0.0135
Calcarine R	0.0103	0.0234	0.0096	0.0067	0.0076	0.0115
Caudate R	0.0116	0.0058	0.0133	0.0061	0.0196	0.0113
Putamen L	0.0009	0.0119	0.0063	0.0027	0.0270	0.0098
Lingual gyrus R	0.0123	0.0034	0.0128	0.0078	0.0068	0.0086
Thalamus L	0.0104	0.0049	0.0036	0.0059	0.0126	0.0075
Caudate L	0.0108	0.0058	0.0096	0.0056	0.0048	0.0073
Precuneus L	0.0107	0.0124	0.0007	0.0060	0.0006	0.0061

*Note*: All brain regions are identified using AAL (automated anatomical labeling). For matrix‐based measures (i.e., Struct M and Func M), the vectors are absolute values of the weights for the connectivity between each brain region and the remaining 89 regions across the different folds of the cross‐validation. For voxel‐wise measures (i.e., GM, WM, ReHo, and ALFF), the vectors are computed using a template mask based on the AAL atlas to extract the absolute value of weight for each brain regions across the different folds of the cross‐validation.

Abbreviations: ALFF, amplitude of low‐frequency fluctuation; Func M, functional connectivity matrix; GM, gray matter; L, left hemisphere; R, right hemisphere; ReHo, regional homogeneity; Struct M, structural covariance matrix; WM, white matter.

## 4 DISCUSSION

In the present article, we aimed to classify patients with schizophrenia and healthy controls by combining sMRI and rs‐fMRI data. In order to assess the reliability of the findings, we used five independent datasets.

Consistent with our first hypothesis, all measures extracted from structural or functional data allowed single‐subject classification with statistically significant accuracy (range: 73.92–83.07%). The finding that both structural and functional measures allow detection of schizophrenia at the level of the individual is consistent with previous studies that used either type of data on its own (J. Liu et al., 2017; Rish & Cecchi, 2017; Schnack et al., 2014; Takayanagi et al., 2011; Venkataraman, Whitford, Westin, Golland, & Kubicki, 2012). However, our investigation extends the results of these previous studies by showing for the first time that functional data allow a higher accuracy of classification than structural data. Interestingly, the single measure that achieved the highest performance of classification was functional connectivity matrix (83.07%). This is consistent with the notion that schizophrenia cannot be explained in terms of localized dysfunction within specific brain areas and is better understood as a disruption of network‐level functional properties (Rish & Cecchi, 2017). In their examination of the relationship between functional and structural brain networks, Wang and colleagues have reported that functional connectivity profiles are largely shaped but not fully determined by structural pathways (Z. Wang, Dai, Gong, Zhou, & He, 2015). This suggests that measures of structural connectivity may be complementary to measures of functional connectivity in the classification of individual patients (Cabral et al., 2016; Mitra et al., 2016). In the present investigation, the use of structural covariance matrix resulted in a mean balanced accuracy of 75.11%. This may confirmed the promising results of previous studies that had investigated this measure in individuals at familial risk for schizophrenia (Tijms et al., 2015) and patients with Alzheimer's disease (Tijms, Moller et al., 2013; Tijms, Wink et al., 2013; Tijms et al., 2014; Tijms et al., 2016).

Consistent with our second hypothesis, within each modality the combination of voxel‐wise images and connectome‐wide based matrices marginally improved performance. Specifically, combining voxel‐wise images and connectome‐wide based matrices within the structural modality improved accuracy to 81.63%, whereas combining voxel‐wise images and connectome‐wide based matrices within the functional modality improved accuracy to 87.59%. To our knowledge, no previous studies have combined voxel‐wise images and connectome‐wide based matrices to detect psychiatric or neurological disorders at the level of the individual. However, we know that the vast majority of psychiatric and neurological illnesses are associated with a combination of the regional and network‐level brain (Fornito & Bullmore, 2015; Worbe, 2015). The results of our investigation, therefore, raise the possibility that the integration of these two types of information might also improve detection in other psychiatric and neurological illnesses.

Different neuroimaging modalities may capture different aspects of neuropathology and therefore may provide complementary information for detecting schizophrenia at the level of the individual patient. Recent studies had shown the advantages of using a multimodal approach for classifying Alzheimer's disease (Dai et al., 2012; D. Zhang, Wang, Zhou, Yuan, & Shen, 2011), Parkinson's disease (Long et al., 2012), PTSD (Q. Zhang et al., 2016) and schizophrenia (Qureshi et al., 2017). Consistent with our third hypothesis, we found that the highest accuracy (90.83%) of classification was achieved when combining all structural and functional measures within a multimodal, multimeasure model. Therefore, combining multimodal measures within a single model appears to be a promising direction for improving classification of individual patients with schizophrenia. However, we note that the higher accuracy of classification resulting from multimodal integration does not necessarily imply clinical utility in real‐world clinical practice. The clinical utility of a clinical test depends on several aspects such as the ability to generate a “divergent prediction” and inform subsequent interventions (Mechelli, Prata, Kefford, & Kapur, 2015). The eventual development of tools for detecting schizophrenia at the level of the individual, therefore, will ultimately require higher levels of diagnostic and prognostic accuracies than those reported in the existing literature. This could be achieved, for example, by combining neuroimaging measures with other types of data using a multivariate supervised ML framework.

In addition, we explored which regions provide the greatest contribution to single‐subject classification. Within the structural modality, the brain regions providing the greatest contribution varied across our three measures (i.e., GM, WM, and structural covariance matrix). In contrast, within the functional modality, we found that the thalamus was among the areas providing the greatest contribution to classification based all three functional measures (i.e., ReHo, ALFF, and functional connectivity matrix). Alterations in thalamic functional connectivity are a key feature of psychotic disorders (Woodward & Heckers, 2016) and include both reduced prefrontal‐thalamic connectivity and increased sensorimotor‐thalamic connectivity (Woodward, Karbasforoushan, & Heckers, 2012). These alterations are also evident in individuals at clinical high risk for schizophrenia, especially those who later go on to convert to psychosis (Anticevic et al., 2015), and therefore are thought to represent a marker of future risk. In addition to the thalamus, the inferior temporal gyrus and middle temporal gyrus contributed to classification in most of our functional measures of interested. The middle temporal gyrus and inferior temporal gyrus subserve a range of cognitive functions, including language processing, semantic memory, and multimodal sensory integration, that are impaired in patients with schizophrenia relative to healthy controls (Kuroki et al., 2006; Onitsuka et al., 2004). Interpretation of these findings must take the multivariate nature of our analytical method into account. While standard mass univariate techniques consider each voxel as a spatially independent unit, multivariate methods such as support vector machine may be additionally based on inter‐regional correlations. An individual region may therefore display high discriminative power due to two possible regions: (a) a difference in volume between groups in that region; (b) a difference in the correlation between that region and other areas between groups. Thus, discriminative networks should be interpreted as a spatially distributed pattern rather than as individual regions. Taken collectively, these findings confirm that both subcortical and cortical networks are implicated in the neuropathology of schizophrenia at the individual level—consistent with current neurobiological models of the disease (Howes & Murray, 2014).

In the present study, when we pooled the five datasets, use site‐stratified cross‐validation and used leave‐one‐dataset‐out cross‐validation, the performance was very poor. This can be explained by the fact that the five datasets were acquired using different scanners and different scanning parameters, resulting in site‐related differences larger than the differences between patients and controls. This aspect of our findings indicates that intersite differences remain a critical challenge in the development of imaging‐based clinical tools and their translational implementation in real‐world psychiatry. Future multisite imaging studies might benefit from the use of novel methods for removing site‐related differences, such as feature harmonization (Xia et al., 2019; Yamashita et al., 2019).

The present study has several limitations. First, our data were acquired at five different sites using different scanners and acquisition parameters; on the other hand, the use of independent datasets allowed us to demonstrate the replicability of our findings. Second, the graph theoretical analysis of sMRI data was implemented using so‐called spatial similarity methods (Kong et al., 2014; H. Wang, Jin, et al., 2016); however, there are alternative graph analytic methods based on intracortical similarity (Tijms, Series, Willshaw, & Lawrie, 2012) that could be used in the future to confirm our findings. In addition, our graphic theoretical analyses were based on the use of Pearson's correlations. Again, there are alternative approaches, such as partial correlation matrices and binary topology metrics, that could be considered in future studies. Third, since our investigation included both structural and functional data, we performed node selection using the AAL atlas, which can be applied to both modalities. Future studies could use alternative approaches such as newly developed functional parcellation (Gordon et al., 2016) to assess the reliability of our findings. Fourth, antipsychotics medication may lead to changes in brain structure (Ho, Andreasen, Ziebell, Pierson, & Magnotta, 2011) and function (Vogel et al., 2016). However, our results were consistent across the five datasets including Dataset 5 in which all patients were medication‐naive; this suggests that our findings are unlikely to be explained by the effects of antipsychotic medication. Finally, a major challenge in the application of ML to high‐dimensional neuroimaging data is the risk of overfitting that is, the learning of irrelevant fluctuations within a dataset that limits generalizability to other datasets. Here we minimized such risk through the use of region‐level features, which are associated with less noise and lower risk of overfitting, rather than voxel‐level data, which are associated with more noise and higher risk of overfitting (Vieira, Pinaya, & Mechelli, 2017). In addition, the fact that our results were replicated in five independent samples, with the use of 10‐fold cross‐validation in each dataset, provides some reassurance about the reliability of the findings.

In conclusion, the present study demonstrates that functional measures allow classification of schizophrenia at the individual level with greater accuracy than structural measures, and that multimodal integration of voxel‐wise images and connectome‐wide based matrices improves accuracy relative to single‐modality classification. These findings are consistent across five datasets with a multimodal model of the disease that includes structural and functional alterations that are expressed at regional and network‐level. We propose that combining multimodal measures within a single model appears to be a promising direction for improving classification of individual patients with schizophrenia. However, the eventual development of clinical tools for detecting schizophrenia and informing treatment will ultimately require higher levels of accuracies than those reported in the present investigation. This might be achieved by combining neuroimaging measures with other types of data within a multivariate supervised ML framework.

## CONFLICT OF INTEREST

The authors declare no competing financial interests.

## Data Availability

Datasets 1 and 2 are available in the public domain, whereas Datasets 3, 4 and 5 are unavailable due to research data confidentiality. This complies with the requirements of the funding bodies and institutional ethics approval.
